# Interactions of marine mammals and birds with offshore membrane enclosures for growing algae (OMEGA)

**DOI:** 10.1186/2046-9063-10-3

**Published:** 2014-05-20

**Authors:** Stephanie N Hughes, Sasha Tozzi, Linden Harris, Shawn Harmsen, Colleen Young, Jon Rask, Sharon Toy-Choutka, Kit Clark, Marilyn Cruickshank, Hamilton Fennie, Julie Kuo, Jonathan D Trent

**Affiliations:** 1 Moss Landing Marine Laboratories, Moss Landing, CA, USA; 2 University of California Santa Cruz, Santa Cruz, CA, USA; 3 University Space Research Association, Moffett Field, CA, USA; 4 California Department of Fish and Wildlife, Santa Cruz, CA, USA; 5 NASA Ames Research Center, Moffett Field, CA, USA; 6 Search for Extraterrestrial Intelligence Institute, Mountain View, CA, USA; 7 CSS-Dynamac Inc., NASA Ames Research Center, Moffett Field, CA 94035, USA

**Keywords:** Biofuels, Wastewater treatment, Photobioreactors, Renewable energy, Marine mammals, Birds, Sea otter, Gulls, Monterey Bay

## Abstract

**Background:**

OMEGA is an integrated aquatic system to produce biofuels, treat and recycle wastewater, capture CO_2_, and expand aquaculture production. This system includes floating photobioreactors (PBRs) that will cover hundreds of hectares in marine bays. To assess the interactions of marine mammals and birds with PBRs, 9 × 1.3 m flat panel and 9.5 × 0.2 m tubular PBRs were deployed in a harbor and monitored day and night from October 10, 2011 to Janurary 22, 2012 using infrared video. To observe interactions with pinnipeds, two trained sea lions (*Zalophus californianus*) and one trained harbor seal (*Phoca vitulina richardii*) were observed and directed to interact with PBRs in tanks. To determine the forces required to puncture PBR plastic and the effects of weathering, Instron measurements were made with a sea otter (*Enhydra lutris*) tooth and bird beaks.

**Results:**

A total of 1,445 interactions of marine mammals and birds with PBRs were observed in the 2,424 hours of video recorded. The 95 marine mammal interactions, 94 by sea otters and one by a sea lion had average durations of three minutes (max 44 min) and represented about 1% of total recording time. The 1,350 bird interactions, primarily coots (*Fulica americana*) and gulls (*Larus occidentalis* and *L. californicus*) had average durations of six minutes (max. 170) and represented 5% of recording time. Interactive behaviors were characterized as passive (feeding, walking, resting, grooming, and social activity) or proactive (biting, pecking, investigating, and unspecified manipulating). Mammal interactions were predominantly proactive, whereas birds were passive. All interactions occurred primarily during the day. Ninety-six percent of otter interactions occurred in winter, whereas 73% of bird interactions in fall, correlating to their abundance in the harbor. Trained pinnipeds followed most commands to bite, drag, and haul-out onto PBRs, made no overt undirected interactions with the PBRs, but showed avoidance behavior to PBR tethers. Instron measurements indicated that sea-otter teeth and gull beaks can penetrate weathered plastic more easily than new plastic.

**Conclusions:**

Otter and bird interactions with experimental PBRs were benign. Large-scale OMEGA systems are predicted to have both positive and negative environmental consequences.

## Background

There is currently considerable interest in the possibility of using microalgae for producing sustainable biofuel, which could provide an alternative to fossil fuels that would not compete with agriculture [1]. Indeed, microalgae species grown on domestic wastewater avoid competing for freshwater and fertilizer [2,3]. Large-scale algae cultivation in the proposed OMEGA system, which grows algae on wastewater and is located offshore, also avoids competing for land [4]. In the OMEGA systems, fast-growing, oil-producing freshwater algae are grown in flexible plastic photobioreactors (PBRs) attached to floating docks, anchored offshore in naturally or artificially protected bays [4,5]. The PBRs use wastewater and CO_2_ from coastal facilities to provide water, nutrients, and carbon for the algae, while the algae contribute to wastewater treatment by removing nutrients as well as toxins and contaminants. The surrounding seawater controls the temperature inside the PBRs and if a PBR module accidentally leaks, the seawater kills the cultivated freshwater algae that might escape. The floating docks that support the OMEGA PBRs may also be used for solar photovoltaic installations and for access to offshore wind turbines and wave generators to produce electricity, as well as to support offshore aquaculture to produce food [6].

Full-scale OMEGA systems that provide energy, process wastewater, and support aquaculture will occupy hundreds or thousands of hectares in protected coastal waters [4]. The environmental impact of such large-scale deployments is unknown, but some of the potential impact may be inferred from studies of harbors, marinas, and offshore platforms [7-10]. These man-made structures are known to have both physical and biological impacts. Physical impacts include changes in local circulation and wave patterns, sediment composition and accumulation rates, as well as light penetration into the water column [11,12]. Biological impacts include changes in local biodiversity and biomass, influencing community structure primarily due to the significant increase in substrate availability [13-18]. Floating structures are also known to form artificial reefs and fish attracting sites [19] and to influence eutrophication by removing excess nutrients and contaminants [20,21]. The changes in these physical and biological factors impact the marine mammals and birds that live in these coastal areas [13,19,22-24].

It is well documented that both offshore and near-shore structures (e.g., piers, breakwaters, docks, wind turbines, oil & gas platforms, and wave generators) impact the behavior and survival of marine mammals and birds [22-27]. On the one hand, these structures are used for resting, feeding, or breeding sites [13,22]; on the other, they pose risks of collisions, entanglement, oiling, acoustic and electromagnetic “noise”, habitat fragmentation, and changes in foraging potential [23-27].

While it seems likely that the OMEGA system will impact marine mammals and birds and conversely that these animals could impact the OMEGA system (biting or pecking, hauling out or perching, and scratching, covering, or fouling surfaces), there are no previous publications about interactions between these animals and floating PBRs.

Here we investigate interactions of marine mammals and birds with PBRs in a harbor by using infrared (IR) video for day and night observations and by conducting experiments with captive pinnipeds. IR video observations were made for 96 days to identify and compare animals interacting with the PBRs and to determine the nature, duration, and timing of their interactions. In experiments with captive, trained pinnipeds, animals in tanks were observed and commanded to bite and haul out onto PBRs to evaluate their responses and to assess their potential for damaging the PBRs. Laboratory experiments were conducted with the tooth of a marine mammal and the beaks of marine birds to determine the forces required to puncture new and weathered PBR plastic, assuming that weathered plastic would be more vulnerable.

These observations and experiments on the coastal marine mammals and birds in the Monterey Bay area provide a basis for understanding how animals react to PBRs and for designing future studies to assess the potential ecological impact of full-scale OMEGA systems.

## Results

### Observations of marine mammal and bird interactions with PBRs

**Figure 1:**
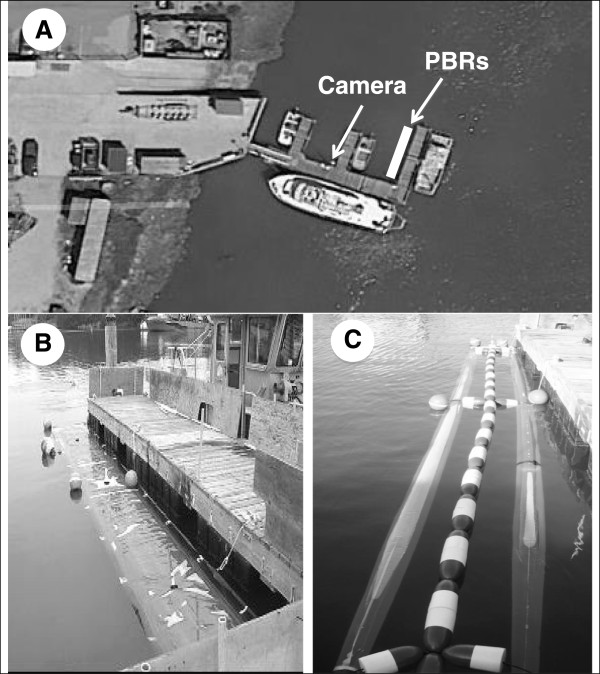
**The experimental site and PBRs in Moss Landing harbor.** The locations of the PBRs and the infra-red video camera **(A)**, which was used to monitor animal interactions with the PBRs day and night. The two PBR types, referred to as flat-panel **(B)** and tubular PBRs **(C)**, were filled with freshwater and had floats attached for added buoyancy.

Nearly 4-months (10 Oct 2011 to 22 Jan 2012) of almost continuous observations were made with IR-video of two types of PBRs (flat panel and tubular) deployed in a boat slip in Moss Landing harbor (Figure 1). The location of the PBRs and the video camera (Figure 1A) as well as the size and appearance of the PBRs themselves are shown (Figure 1B and C). In the 2,424 hours of day and night video, animal interactions were recorded in about 140 total hours (~5.8%), with marine mammals recorded in about 1% of the video and birds in about 4.7%. In general, animal interactions with PBRs were observed mostly during the day and were brief. A total of 1,445 separate interactions were observed between animals and the PBRs, but it was not determined how many individual animals this represents, i.e., it was not determined if these interactions were by many different animals or they were many repeated interactions by a few animals. All interactions were characterized as either passive or proactive (Table 1) and the duration of the interactions was measured from the real-time recordings (Table 2).

**Table 1:** Descriptions of mammal and bird behavior codes

**Code**	**Category**	**Description**
	**Passive**	
1	Feeding	Masticating food items on or in contact with the PBR
2	Walking	Ambulating, crawling, hopping, hauling out (mammals) or flying across PBR (birds)
3	Resting	Perching (birds), sleeping, sitting, or floating motionless on top of PBR
4	Grooming	Preening feathers (birds), or rubbing fur (mammals)
5	Social behavior	Preening, beak fighting, and food parasitism (birds), or vocalization with another animal or group of animals interacting on the PBR
	**Proactive**	
6	Investigative, or potentially harmful	Directly pecking, biting, rolling, or investigating PBR or associated tethers and buoys
7	Unspecified contact, or manipulation	Swimming underneath, or unspecified behavior associated with PBR

**Table 2:** **The animals interacting with PBRs in Moss Landing harbor**, **the nature of the interaction** (**passive or proactive**), **the number of interactions**, **and the duration of the interactions** (**minutes**)

**Interaction**				**Duration**	
**Identity/feeding group**	***Passive***	***Proactive***	**Total**	**Mean ± SE**	**Max**
**Mammals**					
*Invertebrate divers*					
Sea otter (*Enhydra lutris*)	31	63	94	3.1 ± 0.6	44
*Piscivorous cephalopod divers*					
California sea lion (*Zalophus californianus*)	0	1	1	-	1
**Birds**					
*Omnivorous surface feeders*					
American coot (*Fulica americana*)	426	175	601	3.9 ± 0.3	127
Unidentified gull	303	126	429	7.1 ± 0.9	137
Western gull (*Larus occidentalis*)	145	47	192	5.6 ± 1.2	170
Unidentified duck	13	8	21	1.7 ± 0.1	4
Mallard duck (*Anas platyrhynchos*)	0	3	3	-	-
*Carnivorous diving*/*wading feeders*					
Black-crowned night heron (*Nycticorax nycticorax*)	3	1	4	1.5 ± 0.5	2
Double-crested cormorant (*Phalacrocorax auritus*)	2	0	2	-	11
Western grebe (*Aechmophorus occidentalis*)	0	1	1	-	1
Great blue heron (*Ardea herodias*)	2	0	2	6.0 ± 4.0	10
Unidentified cormorant	0	2	2	1	1
*Unidentified birds*	59	34	93	13.0 ± 3.1	190

For marine mammals interacting with the PBRs a single California sea lion interaction was observed in which an animal swam up to a PBR float, interacted and swam away. This interaction was characterized as proactive and lasted about one minute. In contrast, a total of 94 sea otter (*E. lutris*) interactions were observed (Table 2, top). These sea otter interactions were mostly (67%) characterized as “proactive”, indicating the animals were either investigating or manipulating the PBR (see Table 1) and had an average duration of about 3 minutes. One otter however, was observed resting on a PBR, characterized as a “passive” interaction, which lasted about 44 minutes.

There were 1350 bird interactions with PBRs recorded (Table 2, bottom). The interacting birds were divided into three groups: two feeding groups based on their general foraging strategies (omnivorous surface-feeders, carnivorous-diving/wading-feeders) or a general group of “unidentified birds” that were unclear in the video recordings due to either poor image quality or concealed features.

The most frequently observed birds were American Coots (*Fulica americana*), (*n* = 601) and unidentified gulls (*n* = 429), both omnivorous surface feeders. The coots were mostly (71%) passively interacting with the PBRs for average durations of approx. 4 minutes with the longest duration of about 2 hours. Unidentified gulls were also mostly (71%) passively interacting with PBRs with average durations of about 7 minutes and the longest duration of a little over two hours. Also in this category, Western Gulls (*L. occidentalis*) were observed 192 times with mostly passive behaviors (76%), with average interaction durations of about 6 minutes and longest duration of nearly three hours. The other omnivorous surface feeding birds were ducks, which were observed a total of 24 times, about equally characterized as passive or proactive interactions with average durations of about 2 minutes and longest duration of 4 minutes.

Among the carnivorous-diving/wading-feeders the Black-Crowned Heron (*Nycticorax nycticorax*) was seen on four occasions, whereas the other four species were seen only once or twice (Table 2). For carnivorous birds, the Great Blue Heron (*Ardea herodias*) had the greatest average interaction duration of 6 minutes and a longest duration of 10 minutes.

There were 93 observations of “unidentified birds” with 63% of their interactions classified as passive, average duration of 13 minutes and the longest duration of over three hours (Table 2).

In general, the number of interactions with PBRs observed for birds was significantly greater than that of mammals (*χ*^2^_0.05, 1_ = 1086.1, *P* < 0.001), although the PBR interaction duration of sea otters ($\overset{¯}{x}$ = 3.1±0.6, *n* = 94) and birds ($\overset{¯}{x}$ = 5.7±0.4, *n* = 1186) was not significantly different after the data were corrected for outliers to meet the assumption of equal variances [*t* (1259) = 2.056, *P* = 0.22]. There were however, differences in the nature of the PBR interactions.

### Animal behaviors observed in PBR interactions

**Figure 2:**
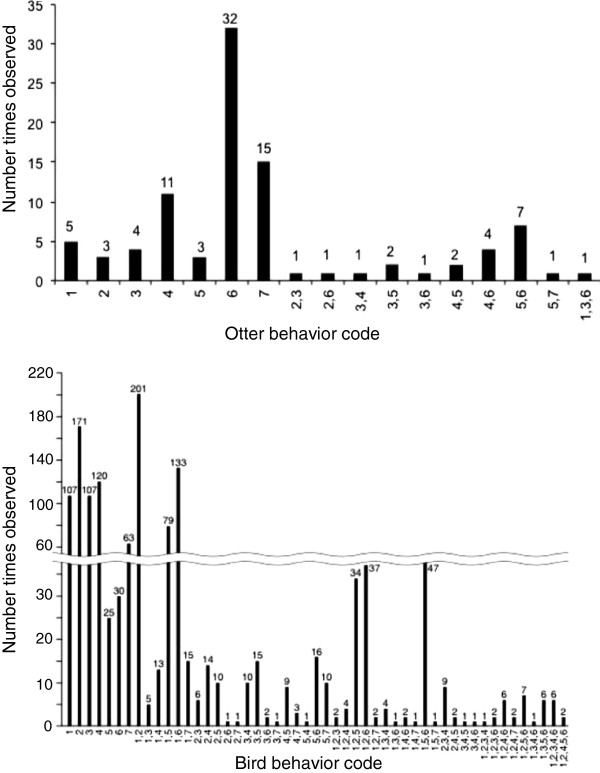
**The number of times different numerically coded behaviors** (**see Table**1**for code definitions**) **or combinations of behaviors were observed for sea otters (top) and birds (bottom).** The numbers at the top of bars are the total number of observations of each behavior.

Passive and proactive behaviors were divided into more detailed behaviors that were numerically coded (see Table 1 for code and Figure 2 for results). Passive interactions and their associated codes were: “feeding” (code 1), “walking” (code 2), “resting” (code 3), “grooming” (code 4), and “social activity” (code 5). Proactive interactions and their codes were: “investigating PBR” (code 6), and “manipulating PBR” (code 7). A single animal could display discreet behavior (single digit codes) or combinations of behaviors (multiple codes).

Sea otters were five times more likely to interact proactively with the PBR than birds (LR, Omnibus *χ*^2^_0.05, 1_ = 53.861, *P* < 0.001), but bird behaviors were more elaborate than those of sea otters. There were 19 behaviors coded for sea otters (Figure 2, top) and there were 50 coded for birds (Figure 2, bottom). The most common sea otter behavior observed was “investigating PBRs” (code 6), which was occasionally combined with “walking” (code 2,6), “resting” (code 3,6), “grooming” (code 4,6), and “social activity” (code 5,6), as well as “feeding + resting” (codes 1,3,6), and “walking + resting” (codes 2,3,6) (Figure 2, top). For birds, the most common behaviors observed were “walking” (code 2) and “feeding + walking” (codes 1, 2), but there were also > 100 observations for resting (code 3), “grooming” (code 4), and “feeding + investigating” (codes 1, 6) (Figure 2, bottom). The observed bird behaviors were more complex than sea otter behaviors as indicated by the code combinations, which for otters used three digits and for birds up to five digits (e.g., 1, 2, 3, 4, 6).

### Time-dependent interactions of animals with PBRs

**Figure 3:**
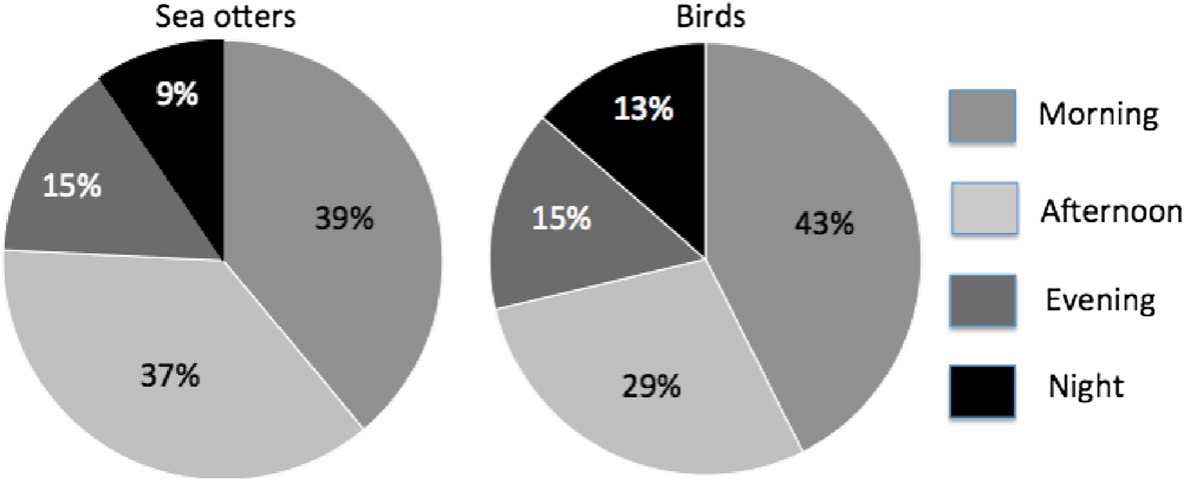
**Percent of animal interactions observed with PBRs in the morning, afternoon, evening, and night for sea otters (left) and birds (right).** Morning was from 0600-1159, afternoon 1200-1759, evening 1800-2359, and night 0000-0559.

Both the sea otters and the birds interacted with PBRs more during the day than in the evening and at night (Figure 3). For the sea otters, 76% of the 94 total observed interactions were in the morning and afternoon with only 24% in the evening and night. Similarly, for birds 71% of the 1350 total observed interactions were in the morning and afternoon with only 29% in the evening and night. For both sea otters and birds this day versus night difference in interactions with PBRs was statistically significant (*χ*^2^_0.05, 1_ = 26.8, *P* < 0.001).

**Figure 4:**
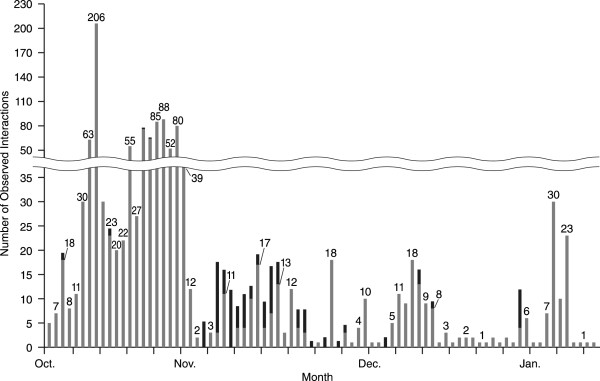
**The number of daily animal interactions with PBRs observed between 10 Oct 2011 and 22 Jan 2012.** Sea otter interactions shown in black and bird interactions in grey. The total number of interactions for each day is shown at the top of each bar.

Whereas sea otters and birds were similar in their day/night interaction with PBRs, their interactions differed significantly with respect to fall vs. winter; i.e., comparing Oct and Nov (fall) with late Nov through Jan (winter) (*χ*^*2*^_0.05, 1_ = 191.5, *P* < 0.001) (Figure 4). Approximately 4% (*n* = 4) of otter interactions occurred in fall, and 96% (*n* = 91) in winter, whereas 73% (*n* = 978) of bird interactions occurred in fall and 27% (*n* = 368) in winter. In Nov, there were 17 days in which multiple otter interactions with the PBRs were observed (Figure 4, middle: black bars). Ten of these days were consecutive and nine of the ten involved multiple observations on the same days. Throughout the study there were bird interactions with the PBRs, but there were distinctly more interactions in Oct. During Oct, there were five or more bird interactions each day with a maximum of 206 interactions observed on Oct 19.

It was not determined if the observed interactions were by the same animals repeatedly visiting the PBRs or if there were many different animals.

### Trained pinnipeds interacting with PBRs

**Figure 5:**
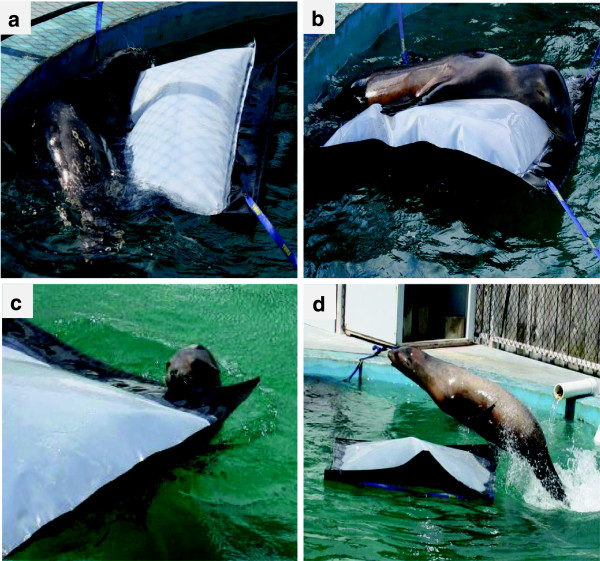
**Trained pinnipeds executing operant commands to interact with PBRs tethered in a tank.** Hauling out onto the PBR by the harbor seal (*Phoca vitulina richardii*) **(a)** and the young sea lion (*Zalophus californianus*) **(b)**, put stress on the PBR welds. Biting and dragging the PBR by a second older sea lion **(c)** and jumping over or onto a floating PBR **(d)** test the durability of its materials and construction.

An experiment was done with one harbor seal and two sea lions to determine (1) if these animals would naturally interact with PBRs, (2) if they would follow commands to do so, and (3) if their interactions would damage the PBRs either from their bites or their weight on the PBR from hauling out or jumping onto them. The animals were introduced separately into a seawater tank with either tethered or floating PBRs that covered about 3% of the surface of the tank (Figure 5). The time each animal was in the tank with the PBRs depended on their response to commands, but the total contact time with PBRs, for all animals, was approximately 1 hour.

The animals gave no indications they were self-motivated to interact with the PBRs, but they were willing to execute trainer commands directing their interactions in exchange for fish rewards. The young sea lion was given the most un-commanded time (approx. 8 min.) to interact with the PBR. It did not notably respond to the float, but its swimming pattern indicated it was responsive to the presence of the PBR tethers and carefully avoided contact with them. The other two animals also avoided the tethers that secured the PBRs.

The observed differences among animals reflected differences in their training and abilities. The harbor seal, despite limited vision from advanced cataracts, consistently fulfilled commands (*n* = 3) to swim under the PBR, rest his head on the PBR, or to haul-out on the PBR (Figure 5a). The younger sea lion responded successfully 89% of the time to these same commands and also to the command to haul out (Figure 5b), to fetch the PBR by biting and pulling (Figure 5c), and to jump over the PBR (Figure 5d). In the “jumping over” behavior the animal usually landed on the PBR and its weight submerged about half of the PBR, which would be expected to stress the welded plastic seams. This young animal also did flipper slaps on the surface of the PBR (not shown) (*n* = 9). The older sea lion was given the same commands as the young sea lion and responded successfully 91% of the time (*n* = 11).

These results indicated (1) these trained pinnipeds did not choose to interact with the PBRs tested unless commanded, but they did avoid swimming into the PBR tethers, (2) innate behavior to ignore the floating PBRs did not outweigh the operant commands to interact with them, and (3) their commanded interactions did not cause damage to the PBRs, although some of the commanded bites, pulls, and hauling-out behaviors had the potential to do damage.

### Force required to puncture PBR plastic

**Figure 6:**
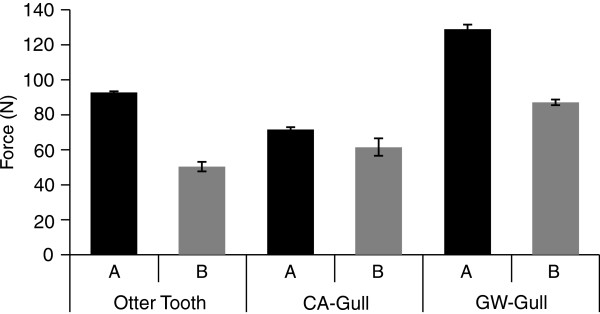
**Force in Newtons (N) required for a sea otter canine tooth (otter tooth) and California gull (CA**-**Gull) or Glaucous**-**winged gull (GW-Gull) beak, to penetrate new (A: black bars) and weathered (B: grey bars) PBR plastic.** The plastic used was clear linear low-density polyethylene LLDPE. The weathered LLDPE was recovered from PBRs that had been exposed, floating in Moss Landing Harbor for 12 weeks. The overall mean puncture force was 78.6 N (±3.7, *n* = 40).

To determine if the linear low density polyethylene (LLDPE) plastic used for the PBRs would be vulnerable to punctures by mammal bites and bird pecks, and to see if this vulnerability changed with plastic weathering, experiments were done with an Instron instrument to determine the forces required to penetrate plastic (Figure 6). Using an otter canine tooth and gull beaks the forces were measured to puncture new LLDPE plastic, which was not exposed to outside conditions (Figure 6A, black columns) and weathered plastic, recovered from PBRs that floated in Moss Landing harbor for 12 weeks (Figure 6B, gray columns). The mean force required for an otter tooth to puncture the new plastic was 92.8 Newtons, (±1.7, *n* = 6) and to puncture weathered plastic was 50.5 Newtons (±1.8, *n* = 7). The mean force required for the California gull beak to puncture new plastic was 71.8 (±2.8, *n* = 7) and weathered plastic 61.5 (±1.2, *n* = 7). The mean force require for the Glaucus-winged gull beak to puncture new plastic was 129.3 N (±5.0, *n* = 4) and weathered plastic was 87.2 (±2.8, *n* = 9). The mean forces required for the otter tooth and gull beaks to puncture new versus weathered plastic were significantly different [*t* (38) = 3.55, *P* = 0.001] as were the mean forces required for the different species to puncture new [*F* (2,14) = 81.7, *P* ≤ 0.001] and weathered [*F* (2,20) =76.88, *P* ≤ 0.001] plastics. While these results indicate that with sufficient force both otter teeth and bird beaks can puncture PBR plastic, particularly after it has weathered, they do not reflect the actual forces produced by the animals.

## Discussion

In coastal environments marine mammals, seabirds, and some aquatic birds opportunistically use floating objects [13,22]. The proposed OMEGA system of floating PBRs and docks will undoubtedly attract marine mammals and birds and influence their behavior, depending on the deployment site. The observations of animal interactions with OMEGA PBRs in Moss Landing harbor reported here provide insights into what may be expected for behavioral modifications of some species of mammals and birds and how these animals may impact PBRs.

The Moss Landing harbor is connected to the Elkhorn Slough, which is adjacent to the productive waters of Monterey Bay and is the third largest estuary in California. The slough is populated by California sea lions, Pacific harbor seals, sea otters and over 250 species of birds [28-32]. Not all species in the slough enter the harbor area, but sea lions, harbor seals, sea otters, and many bird species are frequently observed in the harbor [33-35]. There were many seals and sea lions in the harbor during the study, but only a single brief interaction was observed between a sea lion and a PBR float. In contrast to the pinnipeds, a total of 94 interactions were recorded for sea otters, 805 interactions were recorded for seven identified species of birds and 545 interactions for unidentified birds.

Sea otters are year round residents in Elkhorn Slough and the adjacent harbor, but are most abundant between November and December [36,37], which coincides with the observed peak in their interactions with PBRs. Sea otters were observed resting, grooming, and feeding, on PBRs. They were also observed biting the nylon purge-valves on top of PBRs, rolling on the PBR surface, and investigating or manipulating the high-density foam floats with their paws and mouths. These are all typical sea otter behaviors observed in other contexts and in some cases with man-made objects [31,36,38-40]. Otters in Moss Landing harbor have been observed to haul out on kayaks, boats, and docks (Karl Mayer, pers. comm) and it has been proposed that haul-out behavior may help them conserve energy [41].

All the birds seen interacting with the PBRs are year-round residents in the slough, are commonly seen in the harbor, and in general are most abundant in Monterey Bay in the fall [32,42,43]. Resident gulls and coots nest locally and over-wintering visitors, which can outnumber the resident birds, arrive in early October and stay until mid-November [28]. The increase in the abundance of these birds in October may explain their increased interactions with PBRs during that time. Gulls and coots are omnivores that are known to be well adapted to urbanized environments [44,45] which may explain their opportunistic foraging behavior on the biofouling that developed on the PBRs. The PBR biofouling community in Moss Landing harbor was characterized [46] and found to included diatom films, seaweeds, crustaceans, and bryozoans—known food resources for gulls and coots [28,44]. The observed spikes in the number of coots associated with PBRs may be related to their behavioral tendency to aggregate during foraging [47].

Compared to coots and gulls, other birds (grebes, cormorants, ducks, and herons) were rarely seen on the PBRs probably because the PBRs were outside their normal ambit and there was little foraging attraction. Similarly, there are many shorebirds in the slough (e.g. sandpipers, dunlins, dowitchers, godwits, and willets), which are common on the mudflats adjacent to the harbor [48], but were never observed interacting with PBRs, presumably because the PBRs were out of their preferred habitat.

In general, animal interactions with the experimental PBRs floating in Moss Landing harbor in fall and winter were brief (usually <5 minutes) and all interactions represented <6% of the total video recording time. This means these interactions accounted for a minor portion of the daily activity of the animals in this habitat during this time of the year. Longer studies that include spring and summer are required to determine if there are seasonal behavioral differences that may have more significant impact on the animals or the OMEGA systems. Even these small OMEGA PBRs however, indicated that sea otters and birds will use floating PBRs to forage and rest, suggesting that large-scale OMEGA systems will provide significant foraging areas for marine mammals and many species of birds, depending on the location.

There was no indication that the animals observed in this study, even those that were proactive in their interactions or the captive animals commanded to bite, drag, and jump onto PBRs, damaged the PBRs. While the behavior of trained captive pinnipeds does not reflect what their wild counterparts may do, it does indicate that some behaviors are not problematic for a future OMEGA system of similar design. Instron results indicated that it is possible for otter teeth and bird beaks to puncture PBR plastic, particularly if the plastic was weathered in the harbor for 3 months before testing. The reported biting force of sea otters is proportional to their body size, but even small animals can generate sufficient biting force (>200 N) to puncture the PBR plastic [49]. Although the actual pecking force of gulls was undetermined, for falcons with sharp beaks, it was shown to scale isometrically with body size and for the largest birds it did not exceed 14 N [50]. For granivorous birds, it did not exceed 39 N [51]. These reported pecking forces are less than the 70-90 N measured for the gull beak to puncture the LLDPE plastics even after weathering. Additional experiments are needed to determine if gulls or other birds will be able to peck through PBR plastics or damage them in other ways.

In a full-scale OMEGA deployment, the PBRs and associated support infrastructure will cover hundreds or thousands of hectares of coastal waters in protected bays and is expected to remain in place for years [4]. Depending on the location, such an offshore installation, along with operations for tending, cleaning, and harvesting, is expected to change the local ecology in ways that will impact the marine mammals and birds as well as other coastal community members. In addition to the OMEGA anchoring or mooring systems, which may limit access to foraging areas and create potential entanglement and drowning hazards, there will be changes in the local community due to an increase in sessile and associated organisms on the vast exposed surfaces of OMEGA, and this surface installation will change the water column and benthos due to shading.

It is possible that OMEGA could contribute to plastic pollution in the ocean [52-54] if OMEGA structures and plastics are released into the environment by accidents, harsh weather, or tsunamis. Emergency plans must be developed for OMEGA systems to anticipate and ameliorate such potential environmental problems.

On the other hand, it may be anticipated that the large OMEGA infrastructure will act as a “fish-aggregating device” and become an “artificial reef”, both of which increase local species diversity and expand the local food web [55]. It has been shown that the submerged surfaces of OMEGA PBRs provide substrate, refugia, and habitat for sessile and associated organisms [46], thereby increasing local productivity and diversity and potentially improving coastal water quality [20,21,56].

Observations of coastal marine mammals and birds reported here provide insights into how resident animals react to PBRs deployed within their habitats. Future larger and longer studies should be undertaken in diverse coastal habitats to assess the ecological impacts of full-scale OMEGA systems.

## Conclusion

Given the pressing need for liquid fuels, the importance of sustainable alternatives to fossil oil that do not compete with agriculture [3,57-59], and the evidence that microalgae are the most promising feedstock for such sustainable alternative fuels [60-62], it seems likely that large-scale OMEGA systems will someday be deployed in coastal waters [4]. The OMEGA system uses wastewater from coastal cities, which dispose of their wastewater offshore. To treat the volumes of wastewater from cities and to provide the quantities of biofuels needed, OMEGA installations will cover hundreds or thousands of hectares of coastal waters with PBRs and floating docks. Such structures can be expected to have significant ecological impact in coastal waters. Here, small PBRs were observed day and night for 96 days over a 20-week period. The 94 observed interactions of sea otters and 1350 interactions with birds indicated that even small OMEGA PBRs influence feeding, resting, grooming, and social activity of these animals. In turn, these animals may influence PBRs by biting, pecking, and other manipulations that can damage PBR materials, particularly after they become weathered. While the proposed OMEGA system could provide biofuels, advanced wastewater treatment, and carbon sequestration, as well as a platform for alternative energy and expanded aquaculture, effective coastal management schemes will be needed to accommodate this expanded use of coastal waters [15,63] in preparation for such sustainable systems.

## Materials and methods

### IR-video recordings of marine mammals and birds with PBRs

Nearly continuous video was recorded of PBRs from October 10, 2011 to January 22, 2012 using an infrared (IR) camera (Foscam FI8905W Outdoor Wireless IP Camera, Shenzhen, China). The wireless IR camera was mounted adjacent to the PBRs and filmed 24 hours per day to opportunistically record animal interactions within its field of view. Two PBR designs were observed: flat panel and tubular both of which were made as prototypes by Raven Industries (Souix Falls, South Dakota). The flat-panel PBR (9.5 m × 1.3 m) was made of 0.5 mm translucent linear low-density polyethylene (LLDPE) (top) and opaque black 1.0 mm LLDPE (bottom), whereas the tubular PBR (0.20 m diameter × 9.1 m) was made of 0.38 mm translucent LLDPE (top and bottom). Both PBR designs were filled with freshwater, tethered to high-density foam floats to insure positive buoyancy, and secured alongside the dock at the Marine Operations Facilities of Moss Landing Marine Laboratories (36.804°N, -121.785°W).

The raw video was stored on a hard drive and processed with a video-editing program (Adobe Premiere Pro-CS5; Ottawa, ON, CAN). The video was reviewed at 7× normal speed to mark segments containing marine mammals and birds. These segments were compiled in a separate digital file and viewed in real-time to: (1) identify animals, (2) characterize the nature of the interactions (see categories in Table 1), (3) measure the duration of each interaction, and (4) record the date and time of day (morning, afternoon, evening, or night). When needed, marine mammal and bird experts were consulted to help identify animals. When images were obscure or ambiguous the animals were characterized as “unidentified”. For some analyses, animals were grouped by their foraging strategy.

The nature of the interactions between animals and PBRs was described as either passive or proactive. Passive interactions included behaviors that appeared to be feeding, walking, resting, grooming, or socializing. Proactive interactions were directed toward the PBRs and included pecking, biting, rolling on PBRs, investigating, or behaviors that appeared to be potentially damaging to PBRs. These behaviors were coded as shown in Table 1.

The time of day for each interaction was recorded to the nearest minute and compiled as morning (0600-1159), afternoon (1200-1759), evening (1800-2359), and night (0000-0559). The duration of each interaction and the date were recorded.

### Data analyses

Statistical analyses (PSAW Statistics: 19.0, IMB, USA) were done for data that met the criteria for parametric tests unless stated otherwise. Statistical significance was accepted at α <0.05, and results are expressed as mean ± standard error (SE). Differences in the time and numbers of observed interactions between animals and PBRs were tested using individual Pearson’s chi-squared tests (or Cochran’s chi-squared if *df* = 1). The durations of PBR interaction for mammals were compared to birds using Student’s t-tests provided the interactions were > 30 sec. Interaction durations for the different groups of birds and for mammals were tested using a fixed factor ANOVA. Logistic regression was used to evaluate whether animal group (mammal or bird) accurately predicted passive or proactive interactions with the PBRs. Model fit for logistic regression was assessed using log-likelihood ratio, the Homer and Lemeshow test statistic, and odds ratios to determine the magnitude of effects [64].

### Observations of captive pinnipeds with PBRs

The responses of three captive pinnipeds to model PBRs were investigated in a 7.6 m diameter and 1.7 m deep seawater tank at Long Marine Laboratory of the University of California Santa Cruz. Two female California sea lions (*Zalophus californianus*) that were 2 and 25 years old, and a male harbor seal (*Phoca vitulina richardii*) 23 years old, were observed during three separate sessions over approximately one hour. All three pinnipeds were trained, but naïve to PBR’s prior to testing.

Two custom PBRs (1.5 m × 0.9 m) made by Raven Industries (Sioux Falls, South Dakota) had translucent LLDPE on top and black LLDPE on bottom. They were filled with 114 liters of freshwater to approximate the buoyancy of OMEGA PBRs. For the seal and younger sea lion, one tethered and one free-floating PBR was used. For the older sea lion both PBRs were tethered. To gauge the natural interest of pinnipeds towards PBRs, they were given the opportunity to approach and investigate the PBRs for approximately 8 minutes at the start of the observation session. Subsequently, a trainer commanded each animal to perform operant conditioned behaviors that were previously learned and commonly performed in other contexts. With the PBRs, these trained behaviors included fetching the PBR by dragging it with its teeth, fore-flipper slapping the surface of the PBR, resting its head on the PBR, and jumping over and partly onto the PBR, or hauling-out onto the PBR. These behaviors indicated the willingness and capability of these animals to perform a command and the possible damage they could inflict on PBRs. The responses of each animal were recorded as a success or failure to perform the command and the PBRs were inspected for damage as a result of the interactions.

### Force required to puncture PBR plastic

The force in Newtons (N) required for a sea otter tooth (canine) and three bird beaks to puncture new and weathered PBR plastic (LLDPE) was measured using an Instron 3342 compression testing system (Instron, Norwood, MA). The canine tooth was from a Southern sea otter (*Enhydra lutris*) and the three beaks were from a Glaucous-winged Gull (*Larus glaucescens*) and California Gull (*L. californicus*) from dead, beach-cast animals. Puncture tests were done on samples of new and weathered LLDPE plastic. The weathered plastic was recovered from an OMEGA PBR that was in Moss Landing harbor for 12 weeks. Samples of LLDPE (17.8 cm × 17.8 cm) were secured to a moving force transducer and lowered onto the beak or tooth mounted in the base clamp of the Instron and peak forces were recorded in triplicate, following the manufacturers instructions.

### Instron data analyses

Student’s t-tests were also used for experiments with the Instron to compare the mean forces required for breaking new and weathered plastic with an otter tooth or seabird beaks. Additionally, a fixed factor ANOVA was used to compare the mean forces required for breaking new and weathered plastic by different species.

## Competing interests

The authors declare that they have no competing interests.

## Author’s contributions

JT conceived and developed the OMEGA system including model photobioreactors used in the study. JT, LH, ST, and CY conceived and designed experiments. ST, LH, and ST-C managed data collection and consolidation. SH edited all video data, and MC, HF, and JK gathered behavioral interaction data from the video data. LH gathered data from trained pinniped tank observations. CY provided otter teeth and bird beaks for Instron experiments done by KC, and JR. SNH coded behavioral data, analyzed and interpreted data, and prepared the manuscript with the help of JT. All authors edited and approved the final manuscript for submission to AB.
